# Involvement of RNA-binding protein Hfq in the osmotic-response regulation of *invE *gene expression in *Shigella sonnei*

**DOI:** 10.1186/1471-2180-9-110

**Published:** 2009-05-28

**Authors:** Jiro Mitobe, Tomoko Morita-Ishihara, Akira Ishihama, Haruo Watanabe

**Affiliations:** 1 Department of Bacteriology, National Institute of Infectious Diseases, Shinjuku, Tokyo, Japan; 2 Department of Frontier Bioscience, Hosei University, Koganei, Tokyo, Japan

## Abstract

**Background:**

The expression of Type III secretion system (TTSS) in *Shigella *is regulated in response to changes in environmental osmolarity and temperature. Temperature-dependent regulation of *virF*, the master regulator of TTSS synthesis, is believed to occur at the transcriptional level. We recently demonstrated, however, that TTSS synthesis also involves post-transcriptional regulation of the synthesis of InvE, a target of *virF *and key regulator of TTSS synthesis. The mRNA levels of *invE *(*virB*) are stable at 37°C, but mRNA stability markedly decreases at low temperatures where the TTSS synthesis is tightly repressed. Deletion of *hfq*, which encodes an RNA chaperone in Gram-negative bacteria, results in the restoration of expression of *invE *and other TTSS genes at low temperature due to an increase in the stability of *invE *mRNA. To date, the molecular details of the regulation of TTSS expression in response to osmotic pressure are not known. In the current study, we investigated the mechanism of regulation of TTSS by osmotic pressure.

**Results:**

Transcription of *virF*, which encodes the master regulator of TTSS expression, was partially repressed under low osmotic conditions. Several lines of evidence indicated that osmolarity-dependent changes in TTSS synthesis are controlled at the post-transcriptional level, through the regulation of InvE synthesis. First, the expression InvE protein was tightly repressed under low osmotic growth conditions, even though *invE *mRNA transcripts were readily detectable. Second, under low osmotic conditions, *invE *mRNA was rapidly degraded, whereas deletion of *hfq*, which encodes an RNA chaperone, resulted in increased *invE *mRNA stability and the production of InvE protein. Third, the binding of purified Hfq *in vitro *to *invE *RNA was stronger in low-salt buffer, as assessed by gel-shift analysis and surface plasmon resonance (Biacore analysis).

**Conclusion:**

Osmolarity-dependent changes in TTSS synthesis in *Shigella *involve the post-transcriptional regulation of InvE expression, in addition to partial transcriptional activation by *virF*. The stability of *invE *mRNA is reduced under low osmotic conditions, similar to the effect of temperature. Deletion of an RNA chaperone gene (*hfq*) abolished the repression of TTSS synthesis at low osmolarity through a mechanism that involved increased stability of *invE *mRNA. We propose that the expression of *Shigella *virulence genes in response to both osmolarity and temperature involves the post-transcriptional regulation of expression of InvE, a critical regulator of TTSS synthesis.

## Background

TTSS plays a major role in virulence determination in pathogenic *Shigella*. The expression of TTSS is regulated in response to environmental stimuli, such as changes in salt concentration [1] and growth temperature [2,3]. This response to environmental factors is appropriate for the life cycle of *Shigella*, in which the expression of virulence genes is required for invasion and propagation in the host intestinal tract, but might be a potential burden for survival in the natural environment.

The genes that encode the components of TTSS in *Shigella *are located on the virulence plasmid, and are controlled by two regulator proteins, VirF and InvE (VirB) [4,5]. VirF, an AraC-type transcriptional regulator, activates the transcription of *invE *(*virB*) [4,6-8]. InvE is a homologue of a plasmid-partitioning factor, ParB [7], and possesses DNA binding activity [9]. InvE activates the transcription of the *mxi*-*spa *and *ipa *genes, which encode the components of TTSS, through competition with the global repressor H-NS, a histone-like DNA binding protein [10].

Recently, we reported that the temperature-dependent expression of TTSS is controlled at the post-transcriptional level, through the regulation of InvE synthesis [11]. The mRNA of *invE *is highly stable at 37°C, but stability decreases significantly at 30°C where the TTSS synthesis is tightly repressed. Deletion mutants of *hfq*, which encodes an RNA-binding protein in Gram-negative bacteria, restores the expression of *invE *and other TTSS genes at low temperature due to the increased stability of the *invE *mRNA.

To date, a detailed mechanism of osmolarity-dependent regulation of TTSS expression has yet to be elucidated. In the current study, we examined whether osmotic-dependent changes in TTSS expression involved post-transcriptional regulation. We present several lines of evidence that *invE *expression is regulated at the post-transcriptional level during TTSS synthesis in *Shigella*, and that the RNA chaperone Hfq plays a key role in regulating *invE *mRNA stability.

## Results

### Osmolarity and TTSS expression

The expression of TTSS in *Shigella *is markedly reduced in low-salt LB medium [1]. However, it is not clear whether the critical factor for the decreased expression of TTSS in LB medium is low osmolarity or low-salt concentration. We analysed the expression of TTSS in the presence of several different osmolytes, but similar osmotic pressures. There was a difference in the growth rate of *S. sonnei *in LB medium in the absence (doubling time, 42.1 minutes) and presence (doubling time, 30.6 minutes) of 150 mM NaCl. To control for differences in growth rate in LB medium, we used yeast extract and nutrient broth (YENB) medium [12], since growth rate in YENB in the absence (doubling time, 32.2 minutes) and presence (doubling time, 31.4 minutes) of 150 mM NaCl was similar at 37°C. The osmotic pressure of YENB medium without and with 150 mM NaCl was 96 ± 3 and 397 ± 3 mOsm/kg• H_2_O, respectively. When 150 mM NaCl was replaced with 155 mM KCl, the osmotic pressure was 391 ± 2 mOsm/kg• H_2_O, whereas when NaCl was replaced with 260 mM sorbitol, osmotic pressure was 384 ± 1 mOsm/kg• H_2_O.

**Figure 1:**
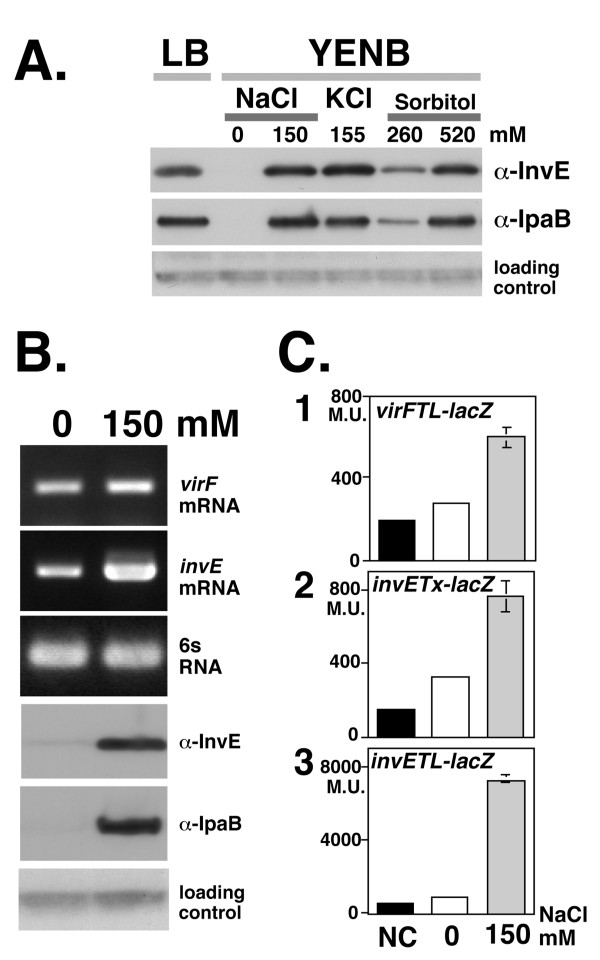
**A. InvE and IpaB expression in different osmotic conditions**. An overnight culture of strain MS390 at 30°C was inoculated into fresh YENB medium with or without osmolytes and then incubated at 37°C until mid-log phase (*A*_600 _= 0.8). Medium, osmolyte, and concentration are indicated at the top of the panel. Antibodies used for detection are indicated on the right of the panels. A cross-reactive unknown protein detected by the anti-InvE antiserum was used as a loading control for InvE Western blot analysis throughout this study. **B**. **Expression of >*invE *and *virF *mRNA and InvE and IpaB protein expression in *S. Sonnei***. Total RNA (100 ng) and 10 μl of the indicate culture were subjected to analysis of mRNA and protein levels, respectively. The 6S RNA *ssrS *gene was used as control for RT-PCR. Primers and antibodies are indicated on the right side of the panels. Concentration of NaCl in the medium is indicated at top of the panel. **C. Expression of *invE *and *virF *>promoter-driven reporter genes**. Wild-type *S. sonnei *strain MS390 carrying the indicated reporter plasmids were subjected to a β-galactosidase assay: Graph 1, *virFTL-lacZ *translational fusion plasmid pHW848; Graph 2, *invETx-lacZ *transcriptional fusion plasmid pJM4320; Graph 3, *invETL-lacZ *translational fusion plasmid pJM4321. Concentration of NaCl is indicated at the bottom of the graphs. Details of the control experiments, indicated by black bars (NC)are described in methods.

To monitor the expression of TTSS, we measured the expression of the effector protein IpaB and the regulatory molecule InvE. The expression of IpaB and InvE was tightly repressed in low osmotic conditions, whereas in the presence of either 150 mM NaCl or 155 mM KCl, the level of both proteins increased to a similar extent (Fig. 1A). A linear relationship was observed between salt concentration and the levels of InvE and IpaB (data not shown), which indicated that there is no threshold for the effective induction of TTSS synthesis. In the presence of 260 mM sorbitol, the levels of both InvE and IpaB were approximately 50% lower than in the presence of NaCl and KCl (Fig. 1A). When the concentration of sorbitol was increased to 520 mM, InvE and IpaB levels increased to the level of the NaCl and KCl growth conditions. These results indicated that in addition to salt concentration, osmolarity regulates the expression of TTSS, although the optimum concentration for maximum induction differed among osmolytes (see discussion).

### Transcription of *virF *and *invE *under low osmotic conditions

Both *ipaB *and *invE *are under the control of the upstream transcriptional regulator VirF [4,6-8]. To identify the level at which IpaB and InvE expression was regulated in response to changes in osmolarity, we analyzed the expression of *virF*. In the absence of salt, *virF *mRNA was detectable by RT-PCR (Fig. 1B, *virF *mRNA), although the level of mRNA expression was approximately 29.0 ± 4.6% of the maximum level observed in the presence of 150 mM NaCl. In an attempt to determine the mechanism of regulation of *virF *transcription, we performed a reporter gene assay in which the expression of *lacZ *was driven by the *virF *promoter [8]. In wild-type *S. sonnei *carrying the *virF-lacZ *reporter gene, the level of β-galactosidase activity in the absence of salt was 20.6% of that in the presence of 150 mM NaCl (Fig. 1C, Graph 1), which indicated that the *virF *promoter is partially active even in the absence of NaCl.

We examined VirF-dependent expression of *invE *by Western blot and RT-PCR. The production of InvE protein was almost completely repressed under conditions of low osmolarity (Fig. 1B, α-InvE), whereas under the same conditions, there was a significant level of *invE *mRNA detectable by RT-PCR (Fig. 1B, *invE *mRNA). Real-time RT-PCR analysis indicated that the amount of *invE *mRNA in the absence of NaCl was 9.5 ± 1.6% of the level in the presence of 150 mM NaCl. We carried out a reporter gene assay to examine the expression of *invE *at both the transcriptional and translational levels [13]. In low osmolarity, β-galactosidase activity in wild-type *S. sonnei *that expressed the transcriptional fusion gene *invETx-lacZ *was moderately decreased, to 28.9% of that seen in the presence of 150 mM NaCl (Fig. 1C, Graph 2). In contrast, β-galactosidase activity in cells that expressed the translational fusion gene *invETL-lacZ *was 7.3% of the level in the presence of 150 mM NaCl (Fig. 1C, Graph 3). These results indicated that the expression of InvE protein is repressed in the absence of salt, a condition under which genes for at least two regulatory proteins are still transcribed, albeit at reduced levels. Thus, the repression of InvE synthesis occurs primarily at the post-transcriptional level.

### Post-transcriptional regulation of *invE*

**Figure 2:**
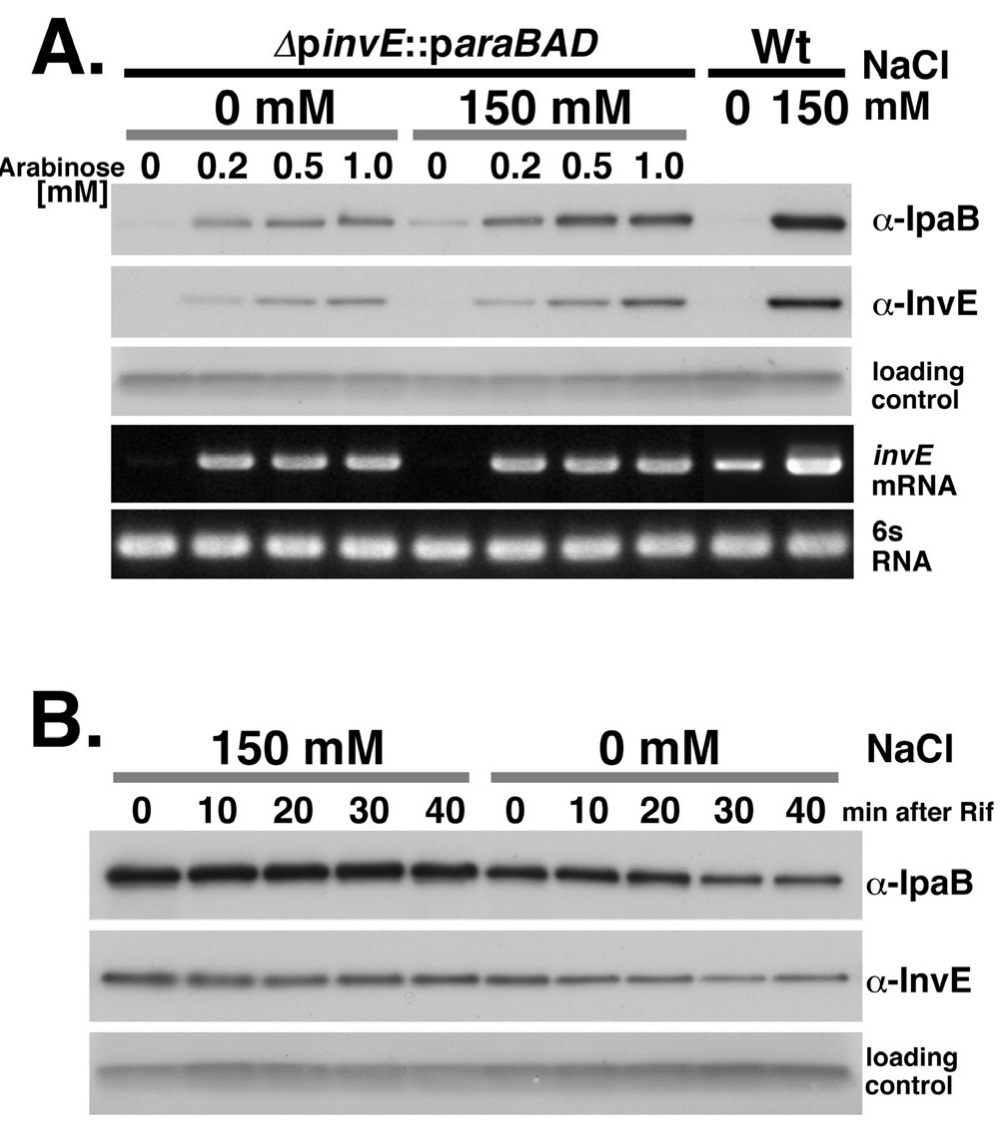
**A. InvE expression in Δp*invE::*p*araBAD *strain MS5512**. Δp*invE::*p*araBAD *strain MS5512 and wild-type strain MS390 were grown overnight in LB medium containing chloramphenicol and 50 μM arabinose, washed twice with fresh LB medium, and then inoculated into YENB media containing increasing concentrations of arabinose and cultured at 37°C with or without 150 mM NaCl, as indicated. Strains (Δ*pinvE::paraBAD*, MS5512; Wt, wild-type strain MS390), concentration of NaCl (0 mM or 150 mM) and concentration of arabinose (0, 0.2, 0.5, 1.0 mM) are indicated above the panels. Primers and antibodies used in the experiments are indicated on the right side of the panels. **B. Stability of InvE protein**. Δ*invE *strain MS1632 carrying the expression plasmid pBAD-invE was grown in YENB media containing ampicillin and 100 μM arabinose, with or without 150 mM NaCl, at 37°C. When cultures reached an *A*_600 _of 0.8, rifampicin was added. Cells were harvested at 10 min intervals for a period of 40 min. Whole cell cultures (10 μl) were analysed by Western blot using anti-InvE and -IpaB antibodies.

To examine the mechanism of post-transcriptional regulation of *invE *expression more directly, we replaced the native *invE *promoter with a promoter cassette containing the *E. coli araC *repressor and the *araBAD *promoter region [14]. In this system, we were able to examine VirF-independent expression of InvE under the control of the AraC-dependent *araBAD *promoter. Strain MS5512 carrying Δp*invE*::p*araBAD *[11] was cultured in the presence or absence of 150 mM NaCl, and the synthesis of InvE protein was induced by increasing the concentration of arabinose. Similar levels of *invE *mRNA were detected in the presence of 0.2 and 1.0 mM arabinose, independently of the presence or absence of NaCl (Fig. 2A, invE mRNA). However, the synthesis of InvE protein was significantly decreased in the absence of NaCl (Fig. 2A, α-InvE), as was InvE-dependent synthesis of the TTSS effector protein IpaB (Fig. 2A, α-IpaB).

To determine whether the low level of InvE protein synthesis under conditions of low NaCl was due to decreased protein stability, we examined the metabolic stability of InvE in an *invE *deletion mutant strain (strain MS1632) carrying an expression plasmid for InvE (pBAD-invE) [11] at various times after treatment with rifampicin. The levels of InvE and IpaB were slightly lower in the absence of NaCl than in the presence of NaCl. Both proteins gradually degraded over time after rifampicin treatment, but the rate of degradation was essentially the same in the presence or absence of NaCl (Fig. 2B). By comparison, *invE *mRNA decayed within 10 minutes (min) after rifampicin treatment, and the rate of decay was much faster in low NaCl than in 150 mM NaCl (see below). These results indicated that InvE protein is metabolically stable once it is synthesized.

### Involvement of Hfq in the post-transcriptional regulation of InvE synthesis

**Figure 3:**
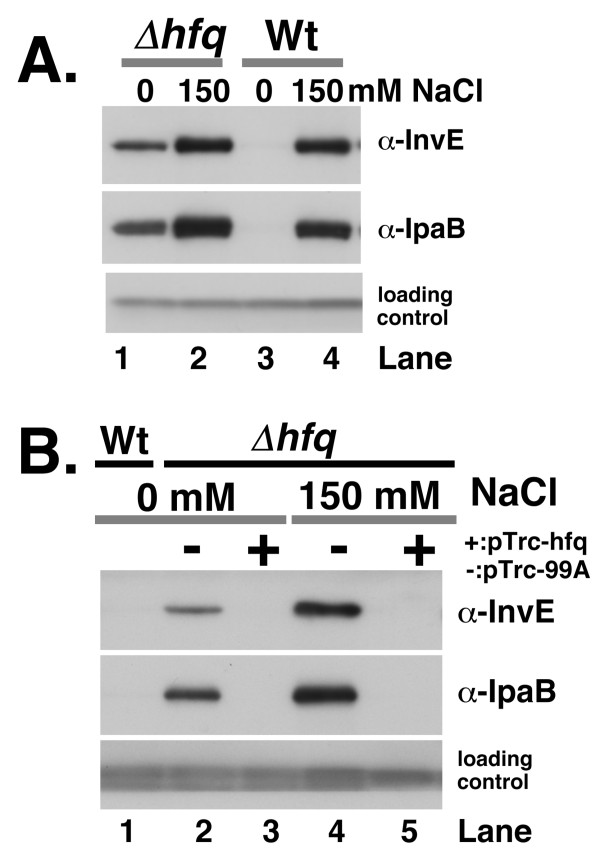
**A. InvE and IpaB expression in the *hfq *deletion mutant**. Wild-type strain MS390 and the *hfq *mutant strain MS4831 were cultured in YENB media with or without NaCl, and then subjected to Western blot analysis. Strains and concentration of NaCl are indicated above the panels. Antibodies used in the experiment are indicated on the right side of the panels. **B. Effect of ectopic Hfq expression on InvE and IpaB in the *hfq *mutant**. *hfq *deletion mutants carrying an Hfq expression plasmid or a control plasmid were subjected to Western blot analysis. Strains were grown in YENB medium containing ampicillin and IPTG, or YENB medium containing ampicillin, IPTG and 150 mM NaCl at 37°C, and then harvested. Strains, concentration of NaCl and plasmids (minus, pTrc99A; plus, pTrc-hfq) are indicated above the panel. Lane 1, wild-type strain MS390 grown in YENB medium; Lane 2, Δ*hfq *(pTrc99A) grown in YENB plus 0.1 mM IPTG; Lane 3, Δ*hfq *(pTrc-hfq) grown in YENB plus 0.1 mM IPTG; Lane 4, Δ*hfq *(pTrc99A) grown in YENB with 150 mM NaCl plus 1 mM IPTG; Lane 5, Δ*hfq *(pTrc-hfq) grown in YENB with 150 mM NaCl plus 1 mM IPTG.

Previously, we showed that the RNA-binding protein Hfq [15,16] is involved in the temperature-dependent regulation of *invE *expression, and that this regulation occurs at the post-transcriptional level [11]. We next examined the expression of InvE in an *hfq *deletion mutant strain of *S. sonnei *(strain MS4831) under low osmotic conditions. As in the case of temperature-dependent regulation, the level of expression of InvE and IpaB in an *hfq *mutant strain in the absence of NaCl was approximately 33% of that seen in the presence of 150 mM NaCl (Fig. 3A lane 1), which suggested that Hfq is involved in the osmolarity-dependent post-transcriptional regulation of InvE and IpaB synthesis. Real-time analysis of *virF *mRNA in the *hfq *mutant in the absence of NaCl indicated that the level of expression of *virF *was 36.5 ± 4.5% of that seen in the wild-type strain in the presence of 150 mM NaCl, which suggested that the level of *virF *transcription in the *hfq *mutant parallels the level of InvE protein synthesis. Thus, in the absence of Hfq, the level of InvE protein in low osmotic conditions correlated with the level of *virF *and *invE *transcription (Fig. 1C, graph 1 and 2). To confirm these results, we introduced an Hfq expression plasmid, pTrc-hfq, into the *hfq *deletion mutant. Ectopic expression of Hfq in the mutant strain resulted in the repression of InvE expression in low osmotic conditions (Fig. 3B, lane 3), and abolished the expression of InvE and IpaB even in physiological osmotic conditions (Fig. 3B, lane 5).

### Stability of *invE *mRNA

**Figure 4:**
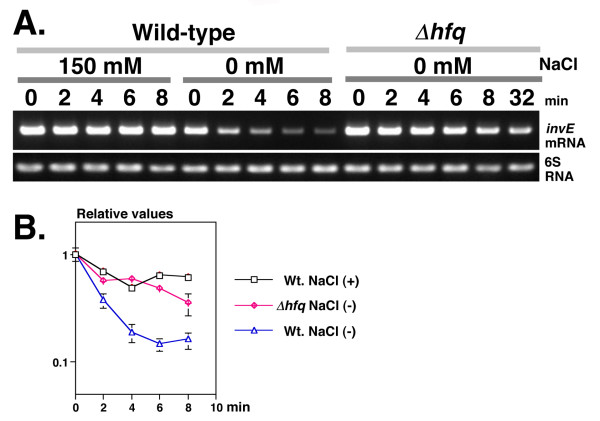
**A. Stability of *invE *mRNA in low osmotic growth conditions**. Pre-cultures were inoculated into 35 ml of fresh YENB media and then grown at 37°C with shaking. When cultures reached an *A*_600 _of 0.8, rifampicin was added, then cells were harvested at 2 min intervals. Total RNA (100 ng) was used for RT-PCR analysis, and 10 μl of the amplified product was subjected to agarose gel electrophoresis. NaCl concentration (150 mM, 0 mM), strains (Wild-type strain MS390; Δ*hfq*, MS4831) and time after rifampicin treatment (0, 2, 4, 6, 8, or 32 min) are indicated above the panels. Primers used in the experiments are indicated on the right side of the panels. **B. Decay curves of *invE *mRNAs**. Total RNA (100 ng) was subjected to real-time PCR analysis. The amount of RNA was normalized to an internal control (6S RNA) and expression was expressed relative to expression at time 0, which was set as 1.0. The X-axis indicates time after rifampicin treatment (0 to 8 min). Presence or absence of 150 mM NaCl (plus, minus) and strains (Wt, wild-type strain MS390; Δ*hfq*, MS4831) are indicated on the right side of the graph.

We examined the stability of *invE *mRNA in the *hfq *mutant by RT-PCR and real-time PCR analysis. Under physiological osmotic conditions, *invE *mRNA levels in the wild-type strain were high, and remained stable for at least 8 min after rifampicin treatment (T_1/2 _= 8.05 min). Under low osmotic conditions, *invE *mRNA levels were low (10 ± 2% of that seen under physiological osmotic conditions), and *invE *mRNA was rapidly degraded within the first 4 min after rifampicin treatment (T_1/2 _= 2.46 min). By comparison, the stability of *invE *mRNA was markedly increased in the *hfq *deletion mutant even under low osmotic conditions (T_1/2 _= 5.70 min) (Fig. 4A and 4B). This increase in *invE *mRNA stability correlated with increased InvE protein levels in cells. These results further support the prediction that the stability of *invE *mRNA is intimately coupled with the expression of InvE protein.

### Hfq-*invE *mRNA interaction *in vitro *under low-salt conditions

In low osmotic conditions, bacteria maintain intracellular osmotic homeostasis through the rapid release of small intracellular molecules, such as ions and amino acids [17]. Since potassium ion is a major cation in bacteria [18], we measured intracellular K^+ ^concentrations in *S. sonnei *under low osmotic conditions. In *S. sonnei *strain MS506 grown in the absence and presence of 150 mM NaCl, the intracellular K^+ ^concentration was 131 ± 4 mmoles/mg cell and 316 ± 0 mmoles/mg cell, respectively. These results indicated that K^+ ^concentration under low osmotic conditions decreases to nearly 40% of that seen under physiological osmotic conditions.

**Figure 5:**
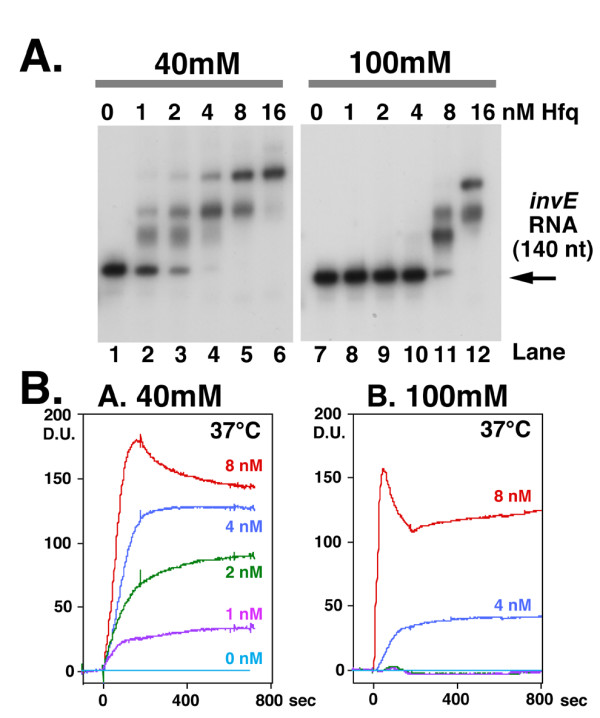
**A. Gel-shift analysis in the presence of 40 mM or 100 mM NH_4_Cl**. A 5'-end labelled *invE *RNA probe (2 nM) was mixed with Hfq protein and then incubated at 37°C for 10 min. Electrophoresis was carried out at 37°C. Concentration of NH_4_Cl (40 mM, 100 mM) and Hfq protein are indicated above the panels. The final concentration of Hfq hexamer was as follows: lanes 1 and 7, 0; lanes 2 and 8, 1 nM; lanes 3 and 9, 2 nM; lanes 4 and 10, 4 nM; lanes 5 and 11, 8 nM; lanes 6 and 12, 16 nM. **B. Analysis of the interaction of Hfq and *invE *RNA by surface plasmon resonance**. The *invE *RNA probe was immobilized onto a sensor chip and binding assays were carried out using a Biacore 2000 optical sensor device. Experiments were performed in 40 mM (Graph A) and 100 mM (Graph B) NH_4_Cl at 37°C. Hfq was diluted in the indicated RNA binding buffer (0, 1, 2, 4 or 8 nM, as indicated on the right side of the graph), and then injected for 180 seconds at a flow rate of 20 ml/min. The results are expressed as difference units (D.U.).

Since interactions between proteins and nucleic acids are influenced by salt concentration, we examined the effect of salt concentration on the interaction of Hfq and *invE *RNA *in vitro*, using an RNA gel-shift assay and surface plasmon resonance (Biacore analysis). Hfq-*invE *RNA complex formation was examined by gel-shift assay using a binding buffer that contained 100 mM NH_4_Cl [19]. To control for the decrease in intracellular K^+ ^concentration in the absence of physiological concentrations of NaCl, we also performed the gel-shift assay in buffer that contained 40 mM NH_4_Cl. The RNA probe (2 nM) was mixed with increasing concentrations of purified Hfq hexamer complex (from 1–16 nM) at 37°C for 10 min. In the presence of 40 mM NH_4_Cl, we observed an initial shift of the RNA probe upon the addition of 1 nM Hfq hexamer (Fig. 5A, lane 1), whereas the corresponding shift in the presence of 100 mM NH_4_Cl required 8 nM hexamer (Fig. 5A, lane 11). The apparent binding constant, as determined by the disappearance of half of the free RNA probe, was 1.7 nM Hfq in the presence of 40 mM NH_4_Cl and 6.2 nM in the presence of 100 mM NH_4_Cl.

We also examined the interaction between Hfq and *invE *RNA by surface plasmon resonance (Biacore analysis). Similar to the gel-shift assay, we examined the interaction in the presence of either 40 mM or 100 mM NH_4_Cl at 37°C. The 140 nucleotide *invE *RNA probe that was used for the gel-shift assay was immobilized onto a sensor chip, and then increasing amounts of Hfq protein were added. The binding of Hfq hexamer to *invE *RNA reached a plateau at a concentration of nearly 8 nM Hfq under both buffer conditions (Fig. 5B) when the Hfq protein was used up to 32 nM (data not shown). Thus, the apparent binding affinity based on surface plasmon resonance was higher than that (16 nM) determined by gel-shift analysis. Distinct differences in the RNA binding properties of Hfq were observed in the presence of 40 mM and 100 mM NH_4_Cl. The minimum concentration of Hfq required for initial binding was 1 nM in the presence of 40 mM NH_4_Cl and 4 nM in the presence of 100 mM NH_4_Cl. In the presence of 40 mM NH_4_Cl, sequential binding of Hfq complexes was observed in an Hfq concentration-dependent manner, whereas in the presence of 100 mM NH_4_Cl, there was a sudden increase in Hfq binding at a concentration of 4 nM Hfq. These results confirmed the results of the gel-shift assay, and indicated that the binding of Hfq to *invE *RNA is influenced by salt concentration.

### Effect of *hfq *mutation on invasion and virulence *in vivo*

To determine whether the repression of TTSS expression in low osmotic conditions influenced invasion by *S. sonnei*, we performed an invasion assay using *S. sonnei *strains that were grown in the absence of NaCl. When grown in low-salt conditions, the ability of the wild-type strain to invade HeLa cells was tightly repressed. The *hfq *mutant strain MS4831 was highly invasive, and invasion was markedly repressed by the addition of IPTG, which induced the expression of Hfq (Table 1). These results indicated that Hfq is intimately involved in synthesis of TTSS-associated genes in *S. sonnei*.

**Table 1:** Invasion efficiency of bacteria grown in low-salt conditions

Bacterial strain	Rate of invasion
HS506	1 ± 1
MS390	2 ± 1
MS4831 (pTrc99A)	100 ± 29
MS4831 (pTrc-hfq)	0
MS390 (YENB+150 mM NaCl)	11 ± 3

In the case of *Shigella, hfq *mutation has been shown to increase invasion efficiency in cultured cell lines [11]. However, *hfq *mutations have also been shown to reduce the virulence of other Gram-negative bacteria in a variety of animal models [20-25] through the regulation of expression of stress response genes [25]. To investigate the role of Hfq in *Shigella *virulence *in vivo*, we performed a Sereny test, in which we monitored the development of keratoconjunctivitis in guinea pigs following inoculation with wild-type and *hfq *mutant strains of *Shigella*.

**Figure 6:**
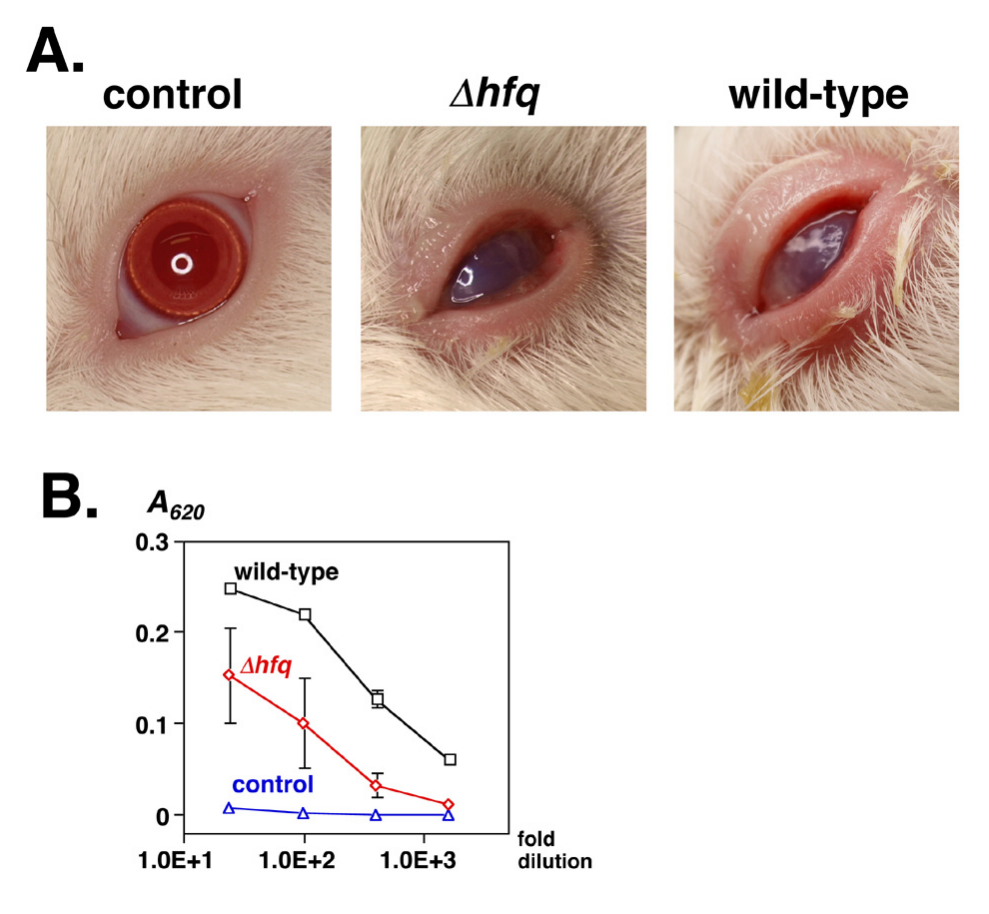
**A. Development of experimental keratoconjunctivitis**. Photograph of the left eyes of guinea pigs 4 days after infection. A bacterial cell suspension (5 × 10^8 ^cells) was dropped into the conjunctival sacs of male Hartley guinea pigs, and the animals were observed for four consecutive days. Left panel, control animal infected with LB medium alone; middle panel, animal infected with Δ*hfq *strain MS4831; right panel, animal infected with wild-type strain MS390. **B. Serum antibodies against effector molecules of TTSS**. Sera were obtained from three animals two weeks after infection. Serial 25-, 100-, 400-, and 1600-fold dilutions were added to immobilized soluble effector molecules (see Methods) on a microtiter plate. Antibodies were detected using peroxidase-conjugated anti-guinea pig IgG. The absorbance at 620 nm (*A*_620_) of each well was monitored after the addition of ABTS using a microplate reader. Black squares, animals infected with wild-type strain MS390; red diamonds, animals infected with Δ*hfq *strain MS4831; blue circles, control animals that received LB medium. Data represents the means and standard deviation of 2 samples.

Guinea pigs infected with either the wild-type or *hfq *mutant strain developed keratoconjunctivitis within three days of infection. The symptoms, including swelling of the cornea, development of conjunctivitis and excretion of pus, appeared to be more severe in animals infected with the wild-type strain (Fig. 6A). The recovery period for animals infected with the wild-type strain was significantly longer on average than for animals infected with the *hfq *mutant strain (8 days versus 5 days, respectively). The production of serum antibodies against TTSS-associated secretary effector molecules was significantly higher in animals that were infected with the wild-type strain (Fig. 6B). Similar results were also observed when using an *hfq *mutant of *S. flexneri *MF4835 (data not shown). Thus, *hfq *mutation appeared to diminish the virulence of *S. sonnei in vivo*, independently of TTSS-associated gene expression.

### Effect of H-NS on *virF *expression in low osmotic conditions

**Figure 7:**
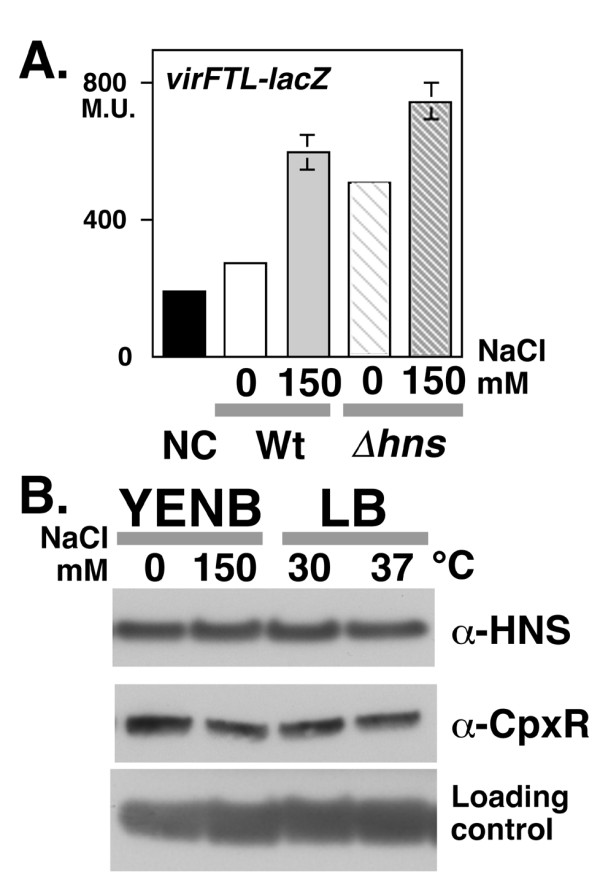
**A. Reporter assay of *virF *promoter activity in an *hns *mutant**. An *hns *deletion mutant of *S. sonnei *strain MS4841 carrying *virFTL-lacZ *(striped bars) was grown in YENB media with or without 150 mM NaCl were subjected to the β-galactosidase assay. For a comparison of activities, the data from Figure 1C, Graph 1, which was derived from simultaneous assays, is indicated by three solid bars on the left side of the graph. Strain and concentration of NaCl are indicated at the bottom of the graph as follows: Wt, wild-type strain (solid bars); *hns*, *hns *deletion mutant (striped bars); YENB medium, 0 (white bars); YENB medium with 150 mM NaCl, 150 mM (gray bars). **B. Western blot analysis of H-NS and CpxR expression**. An overnight LB culture of MS390 at 30°C was inoculated into fresh media and then the cells were cultured until they reached mid-log phase (*A*_600 _= 0.8). Media, temperature (YENB at 37°C; LB at 30°C and 37°C) and the concentration of NaCl are indicated on the top of the panel. Antibodies used for detection are indicated on the right side of the panels. A cross-reactive unknown protein detected by the anti-H-NS antiserum was used as a loding control.

The nucleoid protein H-NS is involved in the expression of TTSS through its ability to regulate *virF *expression [26,27]. The effect of H-NS on *virF *expression in low osmotic conditions was examined using the β-galactosidase reporter gene assay. Although the *hns *mutation of *Shigella *has been reported as transposon insertion, deletion of the full-length *hns *gene resulted in the loss of the virulence plasmid in our experiment using *S. sonnei*. Since the transcription of *virF *is regulated by chromosomal factors, the effect of H-NS on *virF *transcription was examined in the *hns *deletion mutant strain MS4841. In *hns *mutants carrying the *virF-lacZ *reporter gene [8], the β-galactosidase activity under low osmotic conditions was 60.6% of that under physiological osmotic conditions (Fig. 7A). In the *S. sonnei *wild-type strain, it was 20.6% (see Fig. 1C, Graph 1). These results indicated that the nucleoid protein H-NS is involved, at least in part, in the osmolarity-dependent regulation of *virF *expression. The level of H-NS protein and that of the two-component regulator CpxR, which is a critical activator of *virF *transcription [28], were similar under both low and physiological osmotic conditions at 30°C and 37°C (Fig. 7B).

## Discussion

Virulence genes in *Shigella *are expressed in response to increases in temperature and/or osmolarity. Previously, we demonstrated that the temperature-dependent expression of virulence-related genes is regulated mainly at the post-transcriptional level, and that the RNA chaperone Hfq is involved in the translational control of virulence gene mRNA expression [11]. At that time, however, precise details on the mechanism of osmolarity-dependent regulation of virulence gene expression in *Shigella *were unavailable.

The expression and synthesis of TTSS is controlled by the VirF-InvE regulator cascade. The expression of TTSS is markedly reduced by low osmolarity due to the repression of InvE synthesis. In the current study, several lines of evidence indicated that the repression of InvE occurs mainly at the post-transcriptional level: 1) there were significant, albeit low levels of *invE *mRNA in cells under low osmotic conditions, whereas InvE protein was barely detectable (Fig. 1B*invE *mRNA); 2) expression of the translational fusion gene *invE-lacZ *was fully repressed under low osmotic conditions, whereas expression of the corresponding transcriptional fusion gene was only partially repressed (Fig. 1C, Graphs 2 and 3); 3) in an arabinose-inducible promoter system, production of InvE protein decreased under low osmotic conditions even in the presence of sufficient amounts of *invE *mRNA (Fig. 2A); 4) in the absence of the RNA chaperone Hfq, the amount of InvE protein correlated with the level of *virF *transcription, even in low osmotic conditions (Fig. 3A); 5) InvE production was reduced upon over-expression of Hfq protein, even in physiological osmotic conditions (Fig. 3B); and 6) the stability of *invE *mRNA decreased under low osmotic conditions in the wild-type strain, but was increased in the *hfq *mutant (Fig. 4).

The synthesis of TTSS is induced in response to changes in osmolarity. While several osmolytes were able to induce TTSS synthesis, the response was weaker with the non-salt osmolyte sorbitol. Differences in TTSS synthesis in response to different osmolytes might be due to differences in permeability or influx through the bacterial membrane. Under physiological conditions, the contribution of non-salt osmolytes is likely to less relevant, because carbohydrates are almost completely absorbed in the ileum before reaching the colon, where infection and propagation of *Shigella *takes place. In the colon, Na^+ ^ions and water are actively absorbed, and K^+ ^ions are passively secreted, leading to an induction of TTSS synthesis. However, we did not observe significant differences in the expression of TTSS (Fig. 1A) and invasion (data not shown) in the presence of the two ions, which indicates that the trigger for TTSS induction is ionic strength, and not the nature of the ionic species.

In prokaryotes, the regulation of gene expression takes place mainly at the level of transcription. In the expression of a set of genes, however, regulation takes place at any one of several post-transcriptional stages, including the regulation of mRNA stability and translation, through a variety of mechanisms. We propose a model for the post-transcriptional repression of InvE expression in which the association of *invE *mRNA with the RNA chaperone Hfq controls mRNA stability. Recently, it was suggested that an iron-regulated small RNA, RyhB [29], plays a regulatory role in *invE *expression [30]. At present, we cannot rule out the possibility that an interaction between *invE *mRNA and an as-yet unidentified RNA is involved in the temperature- and osmotic pressure-dependent activation of InvE synthesis. To date, various mechanisms have been proposed for the regulation of translation initiation through the modulation of RNA structure, including the structure of the initiation codon [31]. For example, the temperature-dependent formation of a secondary structure within the 5'-untranslated element of the heat-shock operon mRNA of the plant bacterium *Bradyrhizobium japonicum *has been shown to regulate the level of translation of that mRNA [32-34]. In case of *invE *mRNA, a change of the signal that represents thermodynamic alteration of the structure was actually detected in circular dichroism spectroscopy [34] for the 140 nucleotides *invE *RNA [11]. Furthermore, the characteristics of the binding of *invE *mRNA to Hfq in low-salt (Fig. 5) and low-temperature [11] conditions are consistent with an opening of the secondary structure of the RNA through the binding of multiple Hfq molecules. Of note, the pattern of binding of *invE *RNA to Hfq in low-salt buffer was remarkably similar to that seen in low temperature conditions [11]. That indicates that the distribution of RNA-Hfq interaction strength upon the ionic circumstance exists in a similar range, which is defined by the thermodynamic distribution of Hfq binding between 30°C and 37°C. To date, specific molecular sensors of low osmotic conditions or mild temperature change have not been identified. Our results suggest that low osmotic conditions evoke a decrease in intracellular ionic strength, resulting in a similar effect on the strength of the RNA-Hfq interaction as that of decreased temperature. This raises the interesting possibility that post-transcriptional regulation itself represents a sensing system for changes in temperature and osmotic pressure.

The lack of active translation of *invE *mRNA could result in its destabilization [24]. In fact, one of the mechanisms of post-transcriptional regulation is the regulation of mRNA stability [35]. The degradosome is a well-characterized mRNA degradation system that consists of RNaseE, as well as Hfq (46). We examined the role of RNaseE in TTSS synthesis using a deletion mutant (Δ*rne*_701–892_) of the C-terminal region of RnaseE and *E. coli rne-3071*^ts ^strain N3431 [36] carrying expression plasmids for *virF*, *invE *and TTSS genes (pJK1143 and pJK1142, respectively) [4]. TTSS synthesis was unaffected in either of the two strains (data not shown), which indicates that an as-yet unidentified degradation pathway involving Hfq likely plays a role in the degradation of *invE *mRNA.

Similar to other bacterial species, *hfq *mutants of *S. sonnei *and *S. flexneri *exhibited decreased virulence *in vivo*. If the up-regulation of virulence gene expression due to *hfq *deletion leads to efficient antigen presentation for the host immune-system, then the *hfq *deletion is a potentially viable candidate for the development of a more effective *Shigella *vaccine, one that goes beyond the serotype-specific effects seen in current vaccine development [37]. In fact, a *Shigella hfq *mutant is currently under evaluation for use as a vaccine in the guinea pig model [38]. *Shigella *can survive in a range of environmental conditions, such as low osmotic pressure and low temperature, where strict repression of virulence gene expression is required. The development of a bi-functional sensing system for osmolarity and temperature represents an important adaptation for survival by this organism.

## Conclusion

Changes in TTSS synthesis in response to osmotic pressure in *Shigella *involve in part the transcriptional regulation of the master regulator *virF*. In the current study, we demonstrated that post-transcriptional regulation of InvE expression is also involved in TTSS synthesis. This mechanism of post-transcriptional regulation of InvE synthesis was abolished in mutants that lacked *hfq*. The stability of *invE *mRNA was increased in the absence of Hfq, a major RNA chaperone in gram-negative bacteria. We propose that the synthesis of TTSS and pathogenesis of *Shigella *in varying temperature and osmolarity environments is dependent on the post-transcriptional regulation of InvE.

## Methods

### Media, reagents and bacterial culture conditions

Luria-Bertani (LB) medium (LB Lenox, Difco Laboratory, Detroit MI) and YENB medium (0.75% Difco Yeast extract, 0.8% Difco Nutrient broth) [12] were used for the low osmotic media. YENB medium containing 150 mM NaCl (Wako Chemical, Tokyo Japan) was used as the physiological osmotic medium. YENB medium containing 155 mM KCl (Wako) or 260 mM sorbitol (Sigma Co., St. Louis MO) was used as a control for osmotic pressure. The osmotic pressure of each type of medium was measured by the decreasing freezing point method [39] in a clinical inspection facility (SRL Co., Tokyo Japan). The concentrations of antibiotics were as follows: ampicillin (Wako), 50 μg/ml; chloramphenicol (Wako), 12.5 μg/ml; rifampicin (R3501 Sigma), 200 μg/ml. Concentrations are also specified in the Figure legends for each experiment. For all experiments, the indicated strains were inoculated into 2 ml of LB medium and grown overnight at 30°C with shaking (150 rpm) in a water-bath. The cultures were diluted 100-fold in 5 ml of fresh YENB medium with or without salt. The samples were incubated at 37°C with shaking at 150 rpm, and monitored for turbidity at 600 nm (*A*_600_) by spectroscopy (Spectronic™ 20+, Shimadzu Co., Kyoto Japan). Cells were harvested when they reached an *A*_600 _of 0.8. Aliquots of the culture were used for measuring β-galactosidase activity (50 μl), as previously described [40], or subjected to 10% SDS-PAGE and Western blot analysis (10 μl) [41]. The control experiments, indicated by black bars in Figure 1C (NC, negative control), were conducted by Δ*cpxR *strain MS2830 (Graph 1), or strain MS506 cured of virulence plasmid (Graphs 2 and 3) carrying the indicated reporter plasmid. All controls were grown in YENB plus 150 mM NaCl. The percentages indicated in the text were calculated after data was normalized to the negative control. Data represents the means and standard deviation of at least two independent experiments. IpaB and InvE proteins were detected using an anti-IpaB monoclonal antibody and an anti-InvE polyclonal antibody [13], respectively. For the detection of CpxR and H-NS, 5 μl of whole cell culture were separated by 15–20% tricine gradient gel electrophoresis (Wako), and then analysed by Western blot using an anti-CpxR [28] and anti-H-NS antibody, respectively, as previously described [42,43].

### Construction of mutant strains

The bacterial strains and plasmids used in this study are listed in Table 2. Strain MS506 is a tetracycline-sensitive derivative of an avirulent strain, HW506, that was isolated by fusaric acid selection, as described previously [13]. For the construction of a partial deletion mutant of *rne*, we used a PCR-based gene disruption technique and wild-type *S. sonnei *strain MS390. A kanamycin resistant gene cassette in the plasmid pKD13 was amplified with the following primers: rne701us, 5'-GATGATAAACGTCAGGCGCAACAAGAAGCGAAGGCGCTGAATGTTGAAGAGTGAGGCTGGAGCTGCTTCG-3'; and rne701ds, 5'-GCATTTACCGATATGCAGGGATTGTCGCTCTTCCAGCTCAACAAATAATTTCCGGGGATCCGTCGAC-3'. The amplified fragment was inserted into the bacterial chromosome, as described previously [44].

**Table 2:** Bacterial strains and plasmids used in this study

Bacterial strains and plasmids	Genotypes	(references)
E. coli		
N3431	*rne-3071^*ts*^, lacZ43, LAM-, relA1, spoT1 *(CGSC#6975)	[36]
		
S. sonnei		
HW383	*S. sonnei *wild-type strain, (Tc^r^)	[7]
HW506	*S. sonnei *HW383 without pSS120 plasmid (Tc^r^, non invasive)	[7]
MS506	HW506 (Tc^s^)	This study
MS390	HW383 (Tc^s^)	[13]
MS1632	MS390Δ*invE*	[11]
MS2830	MS390Δ*cpxR *(*cpxR*: chromosomal activator of *virF *gene)	[13]
MS4831	MS390Δ*hfq*	[11]
MS4841	MS390Δ*hns *(non invasive)	[11]
MS5400	MS390Δ*rne_701–892_::aphA*	This study
MS5512	MS390Δp*invE*::p*araBAD*	[11]
		
S. flexneri		
2457T	*S. flexneri *2a wild-type strain,	[49]
2457O	2457T carrying mutation in *virF *gene (non-invasive)	[50]
MF4835	2457TΔ*hfq::aphA*	[11]
		
Plasmids		
pBAD-invE	PCR-amplified *invE *gene was cloned into pBAD24 (Ap^r^)	[11]
pHW848	*virF-lacZ *translational fusion plasmid (Cm^r^)	[8]
pJK1142	*invE *and *ipa-mxi-spa *(TTSS) genes encoding plasmid (Km^r^)	[4]
pJK1143	*virF*-encoding plasmid (Cm^r^)	[4]
pJM4320	*invE-lacZYA *transcriptional fusion in pTH18cs5(Cm^r^)	[13]
pJM4321	*invE-lacZYA *translational fusion in pTH18cs5(Cm^r^)	[13]
pTrc99A	IPTG inducible expression plasmid(Ap^r^)	[51]
pTrc-hfq	PCR-amplified *hfq *gene was cloned into pTrc99A(Ap^r^)	[11]

### Measurement of intracellular K+ ion concentration

Intracellular K^+ ^ion concentration was measured by potassium-electrode, as described previously [17]. An avirulent *S. sonnei *strain, MS506, was grown to an *A*_600 _of 0.8 in 45 ml of YENB medium or YENB medium plus 150 mM NaCl at 37°C, and then the culture was chilled on ice for 15 min. The culture was divided into triplicate tubes (15 ml Falcon tubes, #430766, Corning Inc., Corning NY), and then bacterial cells were collected by centrifugation at 5000 × *g *for 15 min at 4°C. An aliquot of each culture was diluted and plated on LB agar for measuring colony counts. The bacterial cells were washed twice at 4°C with 5 ml of hypotonic buffer (20 mM Na-Phosphate pH7.0 for the YENB cultures) or isotonic buffer (20 mM Na-Phosphate pH7.0, 150 mM NaCl for the YENB plus 150 mM NaCl cultures). Cells were suspended in 2 ml of hypotonic buffer and then sonicated using a SONIFIER-250D (Branson Ultrasonic Co., Danbury CT) until microscopic examination confirmed that all the cells were completely disrupted. The samples were cleared by centrifugation at 12000 × *g *for 30 min at 4°C, and the K^+ ^ion concentration of the supernatants was measured by potassium electrode [17] at SRL Co. (Tokyo Japan).

### RNA preparation and detection

Two ml of whole cell culture were quickly mixed with 150 μl of 5% (v/v) water-saturated phenol in ethanol to prevent RNA degradation [45]. *virF *and *invE *mRNAs were purified and analysed using a Titan™ one tube RT-PCR kit (Roche, Indianapolis IN) and Perfect Real-time™ (Takara Bio Co., Shiga Japan), as described previously [11]. For the detection of *virF *mRNA by real-time PCR, virFc-314F (5'-GGAGACGTTTATTTGTATATTTCGCTCTA-3', 120 nM) and virFc-398R (5'-GACGGTTAGCTCAGGCAATGAT-3', 120 nM) primers and the fluorescent probe virFc-345T (5'-FAM-AAAGCAATTTGCCCTTCATCGAT-TAMRA-3', 32 nM) were designed by ABI primer design software (Applied Biosystems Inc., Foster CA) and synthesized by ABI Japan (Tokyo). Real-time PCR analysis was performed using an ABI PRISM 2000 Thermal Cycler, as described previously [11]. RNA preparation and real-time PCR analysis were repeated at least 3 times with similar results.

### Gel-shift assay

The labelled RNA probe (20 fmoles), corresponding to 140 nucleotides of the *invE *gene (starting from the transcription start site at +1) [11], and purified Hfq protein (0, 1, 2, 4, 8, or 16 nM Hfq hexamer) were mixed in a volume of 10 μl in one of two RNA binding buffers (40 mM NH_4_Cl, 10 mM Tris-HCl pH7.5, 5 mM magnesium acetate, 0.1 mM dithiothreitol; or 100 mM NH_4_Cl, 10 mM Tris-HCl pH7.5, 5 mM magnesium acetate, 0.1 mM dithiothreitol) at 37°C for 10 min. Gel-shift analysis was performed at 37°C as described previously [11].

### Surface Plasmon Resonance (Biacore Analysis)

Surface plasmon resonance was performed with Biacore 2000 optical sensor device using the same 140 nucleotide *invE *RNA probe for the gel-shift assay as described previously [11]. The probe was immobilized onto a sensor tip SA (GE Healthcare Co., Piscataway NJ), causing a change of nearly 150 resonance units. Purified Hfq protein was diluted to a final concentration of 0, 1, 2, 4 or 8 nM (Hfq hexamer) in one of two RNA binding buffers, as described for gel-shift assays, and then injected for 180 seconds through two flow cells (flow cell 1, blank; flow cell 2, *invE *RNA) at a flow rate of 20 ml/min at 37°C. Non-specific proteins were dissociated from the chip by washing (for 700 seconds). Bound Hfq protein was subsequently removed with 2 M NaCl. The response value of the reference cell (flow cell 1, blank) was subtracted from the response value of flow cell 2 (*invE *RNA) to correct for nonspecific binding, and the results are expressed as difference units (D.U.). The right panels of Figure 5A and 5B are reprinted from our previous issue [11] with the permission of the American Society for Biochemistry and Molecular Biology (Copyright ^© ^2008), which were performed with the identical materials to the left panels in the same experimental period.

### Invasion assay

Pre-cultures in LB media were inoculated into 5 ml of YENB medium and then incubated for 2 hrs at 37°C with shaking. For strains carrying expression plasmids, IPTG was added to a final concentration of 0.1 mM 40 min after inoculation, and then the cultures were allowed to incubate for an additional 80 min at 37°C. Bacterial invasion into HeLa cells using the gentamicin protection assay was performed as previously described [11].

### Animal experiments

Three groups (6 total) of male Hartley guinea pigs (2 weeks old, SLC Co., Hamamatu Japan) were infected with *S. sonnei *and *S. flexneri *strains for the Sereny test, an experimental animal model of conjunctivitis [46]. Fresh LB cultures of the indicated strains were harvested at an *A*_600 _of 0.8 and then collected by centrifugation. Bacterial cells (5 × 10^8^) in 10 μl of LB medium were deposited into the conjunctival sac of each eye of 2 animals for two consecutive days. Four day later, the symptoms of each animal were recorded by digital photography. Sera were obtained two weeks after infection, and the levels of antibodies against soluble effector molecules of TTSS were measured by ELISA using peroxidase-conjugated anti-guinea pig IgG as the secondary antibody (A5545 Sigma). The source of effector molecules was a culture supernatant of strain MS390 grown at 37°C in LB medium containing 10 μg/ml Congo Red (C6767 Sigma), with which the effector molecules of TTSS are known to be secreted [47]. The culture supernatant (200 μl) was plated onto polystyrene microtiter plates (Costar #3369, Corning) and the plates were incubated at 4°C for 18 hours (hrs). Serial dilutions (25, 100, 400, 1600-fold in phosphate-buffered saline) of guinea pig sera were added to the plate and allowed to react for 2 hrs at 37°C, after which the secondary antibody (5000-fold dilution) was added for 1 hr at room temperature. Absorbance at 620 nm was measured using a Multiskan Ascent microplate reader (Thermo Labsystem, Helsinki Finland) after the addition of 1-Step™ ABTS (2,2'-Azinobis [3-ethylbenzothiazoline-6-sulfonic acid]-diammonium salt) (#37615 Pierce, Rockford IL), as described by the manufacture. All animal experiments were conducted in compliance with the Animal Welfare Act, and adhered to the principles stated in the Guide for Care and Use of Laboratory Animals [48] after approval as #209002-2 by a board of experimental animals at the National Institute of Infectious Diseases (NIDD), Japan.

## Abbreviations

The abbreviations used are: TTSS: Type three secretion system; LB: Luria-Bertani medium; IPTG: isopropyl-1-thio-β-D-galactoside; RT: reverse transcriptase; FAM: 6-carboxyfluorescein; TAMRA: 6-carboxytetramethylrhodamine.

## Authors' contributions

JM carried out the experiments other than invasion analysis. TMI carried out the invasion analysis. AI and HW conceived the study, helped in the biological interpretation, and drafted the manuscript. All authors read and approved the final manuscript.
